# The influence of gene-environment interactions on GHR and IGF-1 expression and their association with growth in brook charr, *Salvelinus fontinalis *(Mitchill)

**DOI:** 10.1186/1471-2156-8-87

**Published:** 2007-12-21

**Authors:** Guillaume Côté, Guy Perry, Pierre Blier, Louis Bernatchez

**Affiliations:** 1 Département de biologie, Université Laval, Québec, Québec, Canada; 2 Department of Biological Sciences, University of Southern California, 3616 Trousdale Pkwy, Los Angeles, CA, USA; 3 Département de biologie et de chimie, UQAR, Rimouski, QC, Canada

## Abstract

**Background:**

Quantitative reaction norm theory proposes that genotype-by-environment interaction (GxE) results from inter-individual differences of expression in adaptive suites of genes in distinct environments. However, environmental norms for actual gene suites are poorly documented. In this study, we investigated the effects of GxE interactions on levels of gene transcription and growth by documenting the impact of rearing environment (freshwater vs. saltwater), sex and genotypic (low vs. high estimated breeding value EBV) effects on the transcription level of insulin-like growth factor (IGF-1) and growth hormone receptor (GHR) in brook charr (*Salvelinus fontinalis*).

**Results:**

Males grew faster than females (μ_♀ _= 1.20 ± 0.07 g·d^-1^, μ_♂ _= 1.46 ± 0.06 g·d^-1^) and high-EBV fish faster than low-EBV fish (μ_LOW _= 0.97 ± 0.05 g·d^-1^, μ_HIGH _= 1.58 ± 0.07 g·d^-1^; p < 0.05). However, growth was markedly lower in saltwater-reared fish than freshwater sibs (μ_FW _= 1.52 ± 0.07 g·d^-1^, μ_SW _= 1.15 ± 0.06 g·d^-1^), yet GHR mRNA transcription level was significantly higher in saltwater than in freshwater (μ_SW _= 0.85 ± 0.05, μ_FW _= 0.61 ± 0.05). The ratio of actual growth to units in assayed mRNA ('individual transcript efficiency', iTE; g·d^-1^·u^-1^) also differed among EBV groups (μ_LOW _= 2.0 ± 0.24 g·d^-1^·u^-1^; μ_HIGH _= 3.7 ± 0.24 g·d^-1^·u^-1^) and environments (μ_SW _= 2.0 ± 0.25 g·d^-1^·u^-1^; μ_FW _= 3.7 ± 0.25 g·d^-1^·u^-1^) for GHR. Males had a lower iTE for GHR than females (μ_♂ _= 2.4 ± 0.29 g·d^-1^·u^-1^; μ_♀ _= 3.1 ± 0.23 g·d^-1^·u^-1^). There was no difference in IGF-1 transcription level between environments (p > 0.7) or EBV groups (p > 0.15) but the level of IGF-1 was four times higher in males than females (μ_♂ _= 2.4 ± 0.11, μ_♀ _= 0.58 ± 0.09; p < 0.0001). We detected significant sexual differences in iTE (μ_♂ _= 1.3 ± 0.59 g·d^-1^·u^-1^; μ_♀ _= 3.9 ± 0.47 g·d^-1^·u^-1^), salinities (μ_SW _= 2.3 ± 0.52 g·d^-1^·u^-1^; μ_FW _= 3.7 ± 0.53 g·d^-1^·u^-1^) and EBV-groups (μ_LOW _= 2.4 ± 0.49 g·d^-1^·u^-1^; μ_HIGH _= 3.8 ± 0.49 g·d^-1^·u^-1^). Interaction between EBV-group and environment was detected for both GHR (p = 0.027) and IGF-1 (p = 0.019), and for iTE in the two genes (p < 0.0001; p < 0.05, respectively), where increased divergence in levels of GHR and IGF-1 transcription occurred among EBV-groups in the saltwater environment.

**Conclusion:**

Our results show that both environment and sex have major impacts on the expression of mRNA for two key genes involved in the physiological pathway for growth. We also demonstrate for the first time, at least in fish, genotype-by-environment interaction at the level of individual gene transcription. This work contributes significantly to ongoing efforts towards documenting environmentally and sexually induced variance of gene activity and understanding the resulting phenotypes.

## Background

An increasing number of studies has documented differential expression of genes induced by environmental change (gene expression plasticity) [1-4]. However, only a handful of them have applied a quantitative genetic framework to document how this phenomenon differs between distinct genotypes, that is genotype-by-environment interaction (GxE) [5,6]. As a fundamental quantitative or qualitative change in gene activity, the physiological genetic basis of GxE interaction to environmental change should be discernable at the molecular level among animals with similar genetic background exposed to different environments [7]. Precise quantification of intracellular processes at the level of individual genes could provide a new insight into events occurring between the gene and the appearance of the trait. Such associations should be particularly effective for genes in physiological pathways known to affect particular phenotypes.

Brook charr (*Salvelinus fontinalis*; Osteichthyes: Salmonidae) life history ranges in habitat from permanent residency in streams or lakes to anadromous incursions [8], where individuals make summer migrations into intermediate-salinity estuaries and coastal marine waters [9,10]. Despite potential tradeoffs with survival [11], annual saltwater migration is considered advantageous because it provides access to superior food resources compared to the freshwater environment, translating into higher final weight-at-age (>4 kg) at the end of the saltwater period and greater longevity (8+ years) compared to freshwater residents (<1 kg and 3–4 years) [9,10]. There is evidence of genetic differentiation between the resident and anadromous life history types, both at the population level [12,13], and as distinct quantitative genetic units within the same population [14]. Exposure to the saline environment should, therefore, involve differentiation in the activity of genes in the growth pathway.

**Figure 1:**
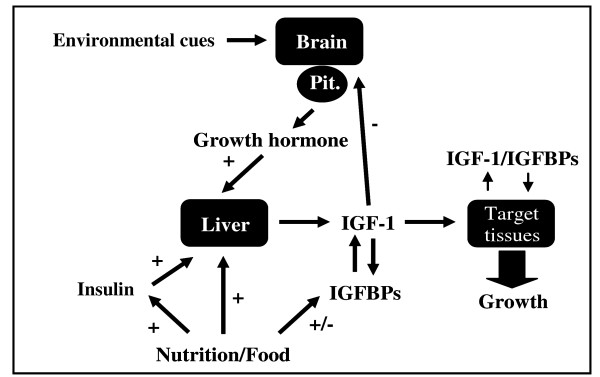
**GH/IGF-1 axis**. Illustration of the endocrine axis controlling growth in teleost fish. Multiple hormonal and nutritional factors may stimulate (+) the production and/or modify (+/-) the activity of IGF-1. Negative feedback (-) by IGF-1 inhibits growth hormone secretion by the pituitary.

As in mammals, growth hormone (GH) and insulin-like growth factor (IGF) are two major molecular targets in the potential endocrine regulation of growth in teleost fishes [15-18]. GH is a pluripotent hormone produced by the pituitary gland in teleosts, and acts by binding to a single-transmembrane receptor, the GH receptor (GHR). Ligand binding induces receptor dimerization producing an active trimeric complex [19]. This active complex stimulates the transcription and production of insulin-like growth factor 1 (IGF-1), a hormone which plays a central role by mediating the growth-promoting actions of pituitary growth hormone [20] (Fig. 1). The direct versus indirect nature of GH action remains to be clarified, but GH appears to act both locally at the target tissue level to stimulate the autocrine/paracrine action of IGF-1, as well as on the liver to increase plasma IGF-1 levels [21]. Expression of both IGF-1 and growth hormone receptor (GHR) mRNA are detected in multiple fish tissues, but expression appears to be greatest in the liver [22]. The impact of environment on expression level of GH/IGF-1 pathway genes is well documented in fish. For instance, work in rainbow trout (*Oncorhynchus mykiss*) [23,24] showed that higher water temperature increases the level IGF-1 and GHR mRNA in liver tissues. Other work on rainbow trout [25] and coho salmon (*O. kisutch*) [26] also demonstrated that the feeding period is associated with increased IGF-1 mRNA expression in muscle and liver, respectively. In several fish species, tissue levels of IGF-1 mRNA positively correlate with body growth rate [19,20,27,28]. Similarly, work with coho salmon [20] and gilthead sea bream (*Sparus aurata*) [29] indicated that the level of hepatic GHR was related to growth performance. However, investment in somatic growth carries inherent metabolic costs and may be limited by energy investment towards maintenance, reproduction and activity, including differential osmoregulation costs between freshwater and saltwater environments [30]. Besides environment and genotypic effects, gene expression may also be influenced by sex. Indeed, differences between sexes in gene activity in the GH/IGF-1 axis have been documented [31-33] and associated with physiological systems (i.e. cortisol receptor, tilapia, [50]). Moreover, it has also been demonstrated that males and females may vary in their transcriptional response to different environmental conditions [34-36].

In this study, we used a quantitative genetic approach to investigate the effects of GxE interactions on levels of gene expression and growth by documenting the impact of rearing environment (freshwater vs. saltwater), sex and genotypic effects on the transcription level of insulin-like growth factor (IGF-1) and growth hormone receptor (GHR) in brook charr. We also predicted that i) differences in transcription level for IGF-1 and GHR should positively correlate with differences in growth, and ii) the production of IGF-1 and GHR should differ between sexes.

## Results

### Freshwater and saltwater growth

**Figure 2:**
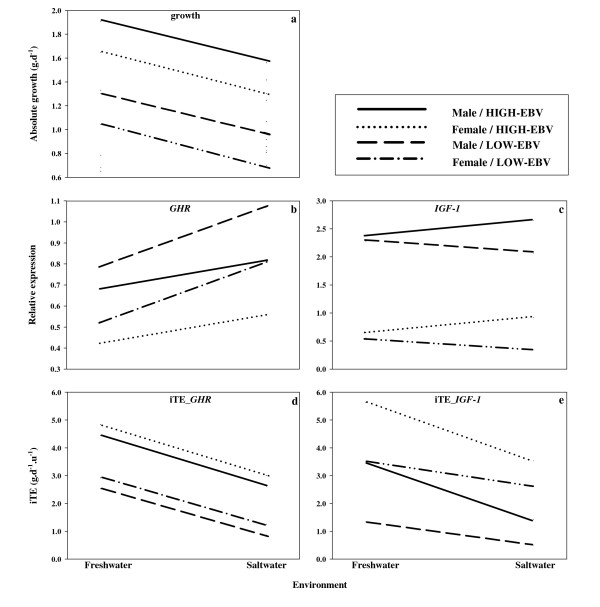
**Reaction norm for growth, gene expression and individual transcript efficiencies**. Predicted linear means for a) absolute growth, b) relative expression of GHR, c) relative expression of IGF-1 and d, e) individual transcript efficiencies (iTE) for GHR and IGF-1 of males and females of HIGH- and LOW-EBV group reared in freshwater and saltwater (20 ppt) environments. Gene-by-environment interaction between EBVgroup-by-environment for growth and iTE_GHR _is not clearly illustrated by non-parallel reaction norms because the difference in EBV group within each environment was small, albeit statistically significant.

We observed marked differences in growth between progeny from the same families in the two salinity conditions (0‰ and 20‰) (Table 1, Figure 2a, see also Table 2 for details on each family). Freshwater-reared individuals grew almost 30% faster than their saltwater-reared full-sibs at the same feeding regime (μ_FW _= 1.52 ± 0.0689 g·d^-1^; μ_SW _= 1.15 ± 0.0572 g·d^-1^). Males had a significantly higher growth rate than females in both environments (μ_male _= 1.46 ± 0.0597 g·d^-1^, μ_female _= 1.20 ± 0.0666 g·d^-1^). HIGH-EBV families also exhibited higher growth (μ = 1.58 ± 0.0663 g·d^-1^) relative to those of the LOW-EBV group (μ = 0.970 ± 0.0537 g·d^-1^). There was also evidence of GxE interactions in the form of variable growth responses between families belonging to different EBV groups in different environments, as detailed below (Table 1).

**Table 1:** Results of the mixed-model maximum likelihood analysis of variance for factors explaining absolute growth (g·d^-1^).

	Absolute growth		
	
Source of variation	d.f	F	*P*
Intercept	143	718.239	<.0001*
Environment	1	22.552	<.0001*
EBV group	1	51.569	0.002*
Sex	1	9.757	0.002*
EBV group × environment	2	19.945	<.0001*

Asterisks indicate significant effects (α = 0.05).

**Table 2:** Absolute growth for male and female of HIGH- and LOW-EBV families reared in freshwater and saltwater environments.

	Absolute growth (g·d^-1^)			
	
	Freshwater		Saltwater	
		
Family	Female	Male	Female	Male
LOW-EBV-1	0.832 ± 0.127	1.518 ± 0.323	0.870 ± 0.187	0.843 ± 0.162
LOW-EBV-13	1.130 ± 0.152	1.517 ± 0.187	0.806 ± 0.138	0.855 ± 0.264
LOW-EBV-20	1.491 ± 0.457	1.291 ± 0.227	0.729 ± 0.162	0.961 ± 0.187
HIGH-EBV-7	1.361 ± 0.205	1.784 ± 0.145	1.244 ± 0.173	1.291 ± 0.187
HIGH-EBV-15	1.712 ± 0.132	2.325 ± 0.264	1.395 ± 0.145	1.901 ± 0.229
HIGH-EBV-24	1.856 ± 0.152	1.096 ± 0.229	1.520 ± 0.264	1.750 ± 0.264

Values are LS means ± SE. LS means were estimated as linear predictors based on the most parsimonious model. Standard errors for each estimate were determined from a bootstrap distribution (1,000 iterations) of model predictions.

### Sex, family and environmental effects on level of gene transcription

GHR level of gene transcription of individuals in the HIGH-EBV group was significantly less (μ = 0.620 ± 0.0819) than that observed in the LOW-EBV group (μ = 0.839 ± 0.0855) (Table 3, Figure 2b; see also Table 4). However, the level of GHR transcription was also significantly higher for fish reared in saltwater (μ = 0.848 ± 0.0460) than their full-sibs reared in freshwater (μ = 0.611 ± 0.0510; p < 0.05), despite the fact that growth in saltwater was slower. There were also marked effects of sex on gene transcription for GHR. GHR expression was almost twice as high in males (μ_♂ _= 0.907 ± 0.0537) than females (μ_♀ _= 0.551 ± 0.0485; p < 0.05). Similarly, IGF-1 transcription level was almost four times higher in males (μ_♂ _= 2.38 ± 0.114) than in females (μ_♀ _= 0.583 ± 0.0910). There was no evidence of environmental effects, or impact of genotypic value, on the expression of IGF-1 (Table 3, Figure 2c).

**Table 3:** Results of the maximum likelihood analysis of variance in mixed models for factors explaining gene expression.

	GHR			IGF-1		
		
Source of variation	d.f.	F	*P*	d.f.	F	*P*
Intercept	145	388.001	<.0001*	145	299.664	<.0001*
Environment	1	12.005	0.001*	1	0.093	0.761
EBV group	1	7.170	0.055*	1	2.444	0.193
Sex	1	13.603	0.001*	1	134.533	<.0001*
EBV group × environment	2	3.689	0.027*	2	4.066	0.019*

Asterisks indicate significant effects (α = 0.05).

**Table 4:** Gene expression of GHR and IGF-1 genes for male and female of HIGH- and LOW-EBV families reared in freshwater and saltwater environments.

	GHR				IGF-1			
		
	Freshwater		Saltwater		Freshwater		Saltwater	
		
Family	Female	Male	Female	Male	Female	Male	Female	Male
LOW-EBV-1	0.687 ± 0.102	1.310 ± 0.260	0.893 ± 0.150	1.09 ± 0.130	0.651 ± 0.217	1.821 ± 0.554	0.557 ± 0.320	1.440 ± 0.277
LOW-EBV-13	0.400 ± 0.123	0.626 ± 0.150	0.697 ± 0.106	1.83 ± 0.213	0.419 ± 0.260	2.611 ± 0.320	0.365 ± 0.226	2.291 ± 0.452
LOW-EBV-20	0.205 ± 0.260	0.679 ± 0.184	0.636 ± 0.130	1.02 ± 0.150	0.196 ± 0.554	2.140 ± 0.391	0.338 ± 0.277	2.530 ± 0.320
HIGH-EBV-7	0.500 ± 0.165	0.579 ± 0.116	0.679 ± 0.139	0.506 ± 0.150	0.489 ± 0.350	2.503 ± 0.248	1.990 ± 0.296	1.784 ± 0.320
HIGH-EBV-15	0.322 ± 0.106	0.549 ± 0.213	0.664 ± 0.116	1.18 ± 0.184	0.495 ± 0.226	2.472 ± 0.452	0.649 ± 0.248	3.362 ± 0.391
HIGH-EBV-24	0.612 ± 0.122	0.861 ± 0.213	0.320 ± 0.184	0.659 ± 0.213	0.577 ± 0.261	2.980 ± 0.452	0.273 ± 0.391	2.670 ± 0.452

Values are LS means ± SE. LS means were estimated as linear predictors based on the most parsimonious model. Standard errors for each estimate were determined from a bootstrap distribution (1,000 iterations) of model predictions.

### Individual transcript efficiency (iTE)

We detected highly significant differences in growth relative to GHR expression among environments with substantially lower gains per unit GHR investment (iTE_GHR_) in fish reared in saltwater (μ_iTE _= 2.00 ± 0.246 g·d^-1^·u^-1^) compared to their freshwater-reared sibs (μ_iTE _= 3.71 ± 0.253 g·d^-1^·u^-1^) (Table 5, Figure 2d; see also Table 6). Females had significantly higher iTE_GHR _(μ_iTE _= 3.12 ± 0.229 g·d^-1^·u^-1^) than males (μ_iTE _= 2.362 ± 0.286 g·d^-1^·u^-1^). Differences among EBV groups for iTE_GHR _were also highly significant where the LOW-EBV group (μ_iTE _= 1.97 ± 0.241 g·d^-1^·u^-1^) showed a much lower effective growth per unit GHR production than the HIGH-EBV group (μ_iTE _= 3.73 ± 0.239 g·d^-1^·u^-1^). Also, there was evidence of interaction between EBV group and environment for iTE_GHR _(see below; Table 5).

**Table 5:** Results of the maximum likelihood analysis of variance in mixed models for factors explaining individual transcript efficiency (iTE).

	iTE_GHR			iTE_IGF-1		
		
Source of variation	d.f.	F	*P*	d.f.	F	*P*
Intercept	146	246.948	<.0001*	146	29.991	<.0001*
Environment	1	60.215	<.0001*	1	6.096	0.014*
EBV group	1	27.786	0.006*	1	5.599	0.077
Sex	1	3.991	0.048*	1	8.699	0.004*
EBV group × environment	2	17.484	<.0001*	2	3.060	0.050*

Asterisks indicate significant effects (α = 0.05).

**Table 6:** Individual transcript efficiency (iTE) of GHR and IGF-1 for male and female of HIGH- and LOW-EBV families reared in freshwater and saltwater environments.

	iTE_GHR				iTE_IGF-1			
		
	Freshwater		Saltwater		Freshwater		Saltwater	
		
Family	Female	Male	Female	Male	Female	Male	Female	Male
LOW-EBV-1	2.509 ± 0.545	1.162 ± 1.39	1.210 ± 0.802	0.804 ± 0.695	3.364 ± 1.112	0.831 ± 2.854	2.620 ± 1.648	0.665 ± 1.427
LOW-EBV-13	3.451 ± 0.655	2.963 ± 0.802	1.380 ± 0.593	0.740 ± 1.135	4.285 ± 1.345	0.588 ± 1.648	2.924 ± 1.217	0.510 ± 2.330
LOW-EBV-20	4.522 ± 1.390	1.891 ± 0.983	1.441 ± 0.695	0.988 ± 0.802	4.194 ± 2.854	0.676 ± 2.018	3.190 ± 1.427	0.379 ± 1.648
HIGH-EBV-7	3.983 ± 0.879	4.272 ± 0.622	2.212 ± 0.743	4.000 ± 0.802	4.880 ± 1.805	4.955 ± 1.276	1.631 ± 1.525	3.920 ± 1.648
HIGH-EBV-15	6.122 ± 0.567	4.823 ± 1.140	2.802 ± 0.622	1.860 ± 0.983	6.310 ± 1.165	0.960 ± 2.330	3.661 ± 1.276	0.574 ± 2.018
HIGH-EBV-24	4.471 ± 0.655	2.032 ± 1.140	3.391 ± 0.983	2.831 ± 1.135	5.365 ± 1.345	0.557 ± 2.330	4.120 ± 2.018	0.701 ± 2.323

Values are LS means ± SE. LS means were estimated as linear predictors based on the most parsimonious model. Standard errors for each estimate were determined from a bootstrap distribution (1,000 iterations) of model predictions.

Similar to findings for GHR, fish in saltwater had significantly lower growth per unit IGF-1 mRNA (μ_iTE _= 2.34 ± 0.516 g·d^-1^·u^-1^) than full-sibs reared in freshwater (μ_iTE _= 3.67 ± 0.530 g·d^-1^·u^-1 ^Table 5 and Figure 2e). Like GHR also, females had significantly higher iTE (μ_♀ _= 3.88 ± 0.471 g·d^-1^·u^-1^) than males (μ_♂ _= 1.28 ± 0.588 g·d^-1^·u^-1^) (Table 5). Likewise, growth per unit IGF-1 was higher in HIGH-EBV (μ_iTE _= 3.78 ± 0.488 g·d^-1^·u^-1^) than in LOW-EBV (μ_iTE _= 2.35 ± 0.491 g·d^-1^·u^-1^) group. We also detected evidence of GxE for iTE_IGF-1 _(see below; Figure 2e).

### Genotype-by-environment interaction

We detected significant gene-by-environment interaction, supported by non-parallel reaction norms (Figure 2), for absolute growth (Table 1), level of gene transcription, as well as individual transcript efficiency for both GHR and IGF-1 genes (Table 3, Table 5). Also, genotype-by-environment interaction within each sex was observed for both GHR and IGF-1, whereby LOW-EBV males produced almost 50% more GHR in saltwater compared to a 20% increase for the HIGH-EBV males. Similarly, LOW-EBV females produced 70% more GHR in saltwater compared to a 20% increase for the HIGH-EBV females (Figure 2b). All EBV groups, however, experienced a net increase in GHR on exposure to saltwater. A similar but reversed association was seen for IGF-1: divergence in transcription level between EBV groups was highest in saltwater and HIGH-EBV individuals produced higher levels of IGF-1 (Figure 2c). In this case, however, both males and females of the LOW-EBV group experienced a net decrease in IGF-1 production.

Net changes in individual weight per unit GHR production (iTE) were approximately parallel among the four sex-EBV groups, although changes in iTE_GHR _among environments were marked (Figure 2d). The iTE_IGF-1 _reaction norms into saltwater were more convergent, so that males and females in the LOW- and HIGH-EBV groups were more similar than in freshwater. Differences in iTE between freshwater and saltwater were more pronounced for HIGH-EBV than LOW-EBV fish, and the increased difference between EBV groups in saltwater was about 30% for females and > 50% for males (Figure 2e).

## Discussion

The aim of this study was to determine the impact of interactions between environments, sexes and genetic values (EBV) on the transcription level of two key genes in the GH/IGF-1 axis and their association with growth in brook charr. Our results provided evidence of GxE interactions for individual gene transcription, as well as pronounced sex effects. We observed that freshwater-reared individuals grew almost 30% faster than saltwater-reared full-sibs under the same feeding regime. A first explanation for such differences could be intolerance of moderate salinity by the Rupert R. charr population, either as a failure of saltwater acclimation, or because of inappropriate genetic architecture for saltwater tolerance. Studies on Atlantic salmon (*Salmo salar*) [37] and kokanee (*O. nerka*) [38] showed that physiological shifts from non-anadromy to anadromy are possible for populations confined to freshwater for thousands of generations. There is also a well documented case of the adoption of anadromy in a population of introduced freshwater rainbow trout (*O. mykiss*) [39]. Therefore, we expected *a priori *that similar plasticity would apply to brook charr from the Rupert R. In contrast, our results suggest that the capacity for osmoregulatory efficiency in saltwater may not be ubiquitous among resident populations of salmonid species, and that this capacity may have been partially lost in the Rupert R. brook charr population. Secondly, we cannot completely refute the possibility that Rupert R. brook charr grew more slowly in the saltwater environment because of the ration used in this study (0.8% body weight/d). A more extensive range of feeding regimes was not feasible due to space constraints. Consequently, a single standardized commercial ration level was chosen for ensuring equal feeding opportunity to all members of the same tank/treatment group, and to avoid confounding effects of growth with excess feed availability. Thus, while this study revealed important sexual, genetic and environmental effects on the activity and efficiency of the genomic transcripts for the same diet, poor mechanical physiological ability might be compensated for through increased consumption rate. This, however, remains to be experimentally tested. We also observed that females had lower overall growth than males, but no evidence of sex-by-environment interaction for growth specifically. The higher overall growth for males compared to females could be the result of differences in bioenergetic costs associated with sexual maturation. Given that our sampling was conducted during late summer and early fall, the period immediately preceding spawning, it is plausible that females were allocating proportionally more energy to gonadal tissues, and less to skeletal growth, than males [40-42].

Many studies have documented the effects of the environment on levels of gene expression [23,24,43,44]. Our results add to these studies by revealing differential gene expression under distinct environments in the brook charr. However, they refuted our working hypothesis since we observed that fish reared in saltwater expressed more GHR mRNA, despite the fact that they had a slower growth rate than fish reared in freshwater. As a consequence, effective growth relative to transcript production – individual transcript efficiency (iTE) – was also considerably higher in freshwater-reared individuals than their saltwater-reared siblings for both genes. At any given point during their development, organisms must invest energy into basal and active metabolism, reproduction, catabolic processes and stress resistance. Our findings suggest that the relative investment to produce the same amount of tissue in saltwater-reared brook charr is considerably higher than in the freshwater environment, particularly so for GHR. This suggests either an environmental inhibition of molecular elements of growth below GHR and/or a failure of control in the production of GHR and/or precursors to it in saltwater. The direct ratio of growth with gene production on the g-per-unit mRNA scale implicit in the iTE index assumes proportionality and uniformity in pathways of the GH/IGF-1 system candidate genes themselves and in the surrounding metabolic systems along the translational and transcriptional axes. As such, the ratio is analogous to a single-gene assay just above the level of candidate gene analysis, or of a single-QTL effect on phenotypic variance observed in contrasting environments which partitions phenotypic variance into locus-specific effects without knowledge of all physiological processes occurring between genetic and phenotypic expression. While our family-replicated design should have sufficiently accounted for most sources of background variance, we cannot exclude the possibility that full-sib means were partially mediated by dominant genetic effects which may partially bias estimates of additive variance [45,46]. Future studies should emphasize a more complete analysis with a full range of physiological products of the growth pathway and associated members. However, the genes analyzed here are ubiquitous in the process of growth, and represent relevant candidates for the description of this pathway.

Partial discrepancies between the transcription level of genes associated with GH/IGF-1 and growth rate may have several physiological explanations. The GH/IGF-1 system has long been recognized as an important participant in the osmoregulatory physiology of fishes, at least for euryhaline species [21,22]. The absence of differences for the transcription level of liver mRNA IGF-1 between salt and freshwater has previously been observed in rainbow trout, for which an abrupt transfer to 80% seawater resulted in increased IGF-1 mRNA levels in gills and kidneys, but not in the liver [47]. In contrast, and similar to our observations for GHR, Sakamoto and Hirano [48] showed that rainbow trout acclimated to seawater experienced an initial decrease in liver GHR followed by a significant increase after four days. Thus, our results, and those of Sakamoto and Hirano [48], indicate the likelihood of at least partial mediation by the liver in seawater adaptation. Unlike Sakamoto and Hirano [48], however, we did not evaluate the level of active GHR in the membrane, but rather estimated the number of copies of the gene product. This suggests that the density of the receptor at the cell surface may depend not only on the rate of gene expression and the stability of the GHR mRNA, but also on the removal of the protein [21].

We also detected a sex effect on gene activity in which the expression of IGF-1 and GHR was significantly higher for brook charr males. Differences between sex in IGF and GH production have also been reported in other species, such as chickens [49,50], and pigs [31], where males typically have higher production rates than females, as in this study. Similarly, these authors observed that male growth per GHR production was actually lower than that of females, which also corroborates our results. Sex steroid hormones may influence the GH/IGF-1 axis. Riley *et al*. [33] found that in sexually dimorphic tilapia, injection of 17β-estradiol into males resulted in a GH/IGF-1 profile more closely resembling that of female fish (namely a lower plasma level of IGF-1 and higher plasma levels of GH), whereas administering di-hydrotestosterone to females elicited a serum GH/IGF-1 profile resembling that of males. From these observations [33,51], Wood *et al*. [22] suggested cross-involvement between sex steroid hormones and the GH/IGF-1 axis, possibly at the level of hepatic GHR. GH resistance induced by sex steroids may, thus, contribute to the developmental switch between somatic and reproductive development associated with sexual maturation in fishes.

Genotypic effect on gene expression, measured as the difference between EBV groups, was observed for both GHR and IGF-1. This corroborates previous observations (e.g. *Drosophila melanogaster*, [52]; *Fundulus heteroclitus*, [53]) that the genotype has a significant impact on variation in gene expression between individuals within population. To our knowledge, however, our study represents one of very few reports of a GxE interaction for levels of gene expression [5,6], whereby the effect of genotypic value on gene expression changed in relation to environmental condition. As such, non-parallel reaction norms for expression of GHR and IGF-1 genes indicate genetic variance in reaction norm for growth at the level of individual genes in the GH/IGF-1 axis as a function of the environment. Given its link with fitness, genetic variation for growth could be maintained by a form of balancing selection: the presence of GxE interaction for individual genes of the GH/IGF-1 axis can change the genetic target for selection because different genotypes may produce optimal phenotypes under different environmental conditions. Both mutations and environmental shock during developmental processes [54,55], as well as quantitative genetic variance for environmental reaction, have been proposed for explaining phenotypically plastic responses to different environments [56,57].

## Conclusion

In summary, we documented that both environment and sex had major impacts on the expression of mRNA for two key genes, GHR and IGF, involved in the physiological pathway for growth. We also demonstrated for the first time, at least in fish, genotype-by-environment interaction at the level of individual gene transcription. As such, this work contributes significantly to ongoing efforts towards documenting environmentally and sexually induced variance of gene activity, and to understand the resulting phenotypes. However, to achieve a more complete understanding of the molecular architecture responsible for the variation of a quantitative trait such as growth, future research should quantify both gene expression and the resultant proteins implicated in the physiological pathway underlying the trait. The continued increase in the use of methods such as RT-qPCR and microarrays in different fields of biology and physiology should greatly improve our understanding of the functional and evolutionary significance of variation in gene expression.

## Methods

### Husbandry and strain history

In 2001, 20 mixed half-sib families were generated at the Laboratoire Régional des Sciences Aquatiques (LARSA, Université Laval, QC, Canada) from 15 sires and 10 dams (Table 7) originating from a strain of brook charr derived from the Rupert River which drains into the James-Hudson Bay in northwestern Québec. Individuals, marked by external T-tags (Floy Inc., Seattle, Washington, USA), were randomly divided and assigned to one of two re-circulation units, each composed of three 3,000 L tanks. All fish were maintained at 10°C and 90% oxygen saturation, and on external photoperiod (50°C25'N, 73°53'W, Québec, CA). Fish were fed 0.8% of their body weight (commercial feed pellets, Corey Feed Mills, Inc., NB) throughout the experiment. At the end of June 2004 (2+ age class), salinity was increased in one of the units (20‰) (SW; n = 415) while being maintained at freshwater (≈0‰) (FW; n = 433) in the other for a period of five months. This salinity is typical of conditions commonly encountered by brook charr in estuarine and coastal marine waters.

**Table 7:** Sample information.

Dam	EBV	Sire	EBV	Family	*n*	EBV Group
**126**	**-0.253**	**196**	**-0.205**	**1**	**102**	**LOW**
126	-0.253	100	-0.042	2	52	
126	-0.253	162	-0.204	3	78	
193	0.029	162	-0.204	4	88	
193	0.029	100	-0.042	5	90	
193	0.029	138	-0.178	6	82	
**252**	**0.184**	**88**	**0.276**	**7**	**90**	**HIGH**
252	0.184	249	0.313	8	84	
252	0.184	196	-0.205	9	79	
252	0.184	138	-0.178	10	13	
**217**	**-0.262**	**250**	**-0.262**	**13**	**102**	**LOW**
**242**	**0.218**	**84**	**0.185**	**15**	**93**	**HIGH**
251	0.159	177	0.081	16	44	
231	-0.049	114	-0.049	17	93	
**38**	**-0.188**	**247**	**-0.197**	**20**	**27**	**LOW**
38	-0.188	148	0.185	21	134	
38	-0.188	177	0.081	22	127	
**94**	**0.212**	**148**	**0.185**	**24**	**88**	**HIGH**
135	-0.048	177	0.081	26	55	
135	-0.048	235	0.030	27	84	

Rupert strain pedigree, estimated breeding value (EBV) for each dam and sire, number of individuals and family selected in reference to HIGH- and LOW EBV group (in bold characters).

### Growth measurements and tissue sampling

Absolute growth (GA = ((W2-W1)/d), where W1 and W2 represent weight for the two successive samplings and d represents the interval in days [10] were calculated for the entire population for the period of July to October. In November 2004, 6 – 15 fish were selected at random within each half-sib family (see below) in the freshwater and saltwater treatments. Fish were killed by rapid decapitation and ≈200 mg of liver tissue was removed and immediately frozen in liquid nitrogen for subsequent analysis of gene expression by reversed transcribed quantitative PCR (RT-qPCR). Individuals were sexed by examination of the gonads at euthanization.

### Quantitative genetic modeling

We used a reduced animal model [58-61] for the estimation of additive quantitative genetic variance (σ2a) and best linear unbiased predictions (BLUP; [58]) of breeding value (estimated breeding value; EBV) for absolute growth for all individuals (Perry et al., unpublished). Parent-offspring relationship and growth rate (as a single-vector phenotype) were coded using PEST [62] for REML in VCE5.1 [61] with the iteration of analytical gradients [60] in the animal model

y = Xβ + Za + e

where y is the phenotypic vector for growth rate, X is the design matrix (n × p) of fixed effects, β the fixed effects coefficient vector (p × 1), Z is the incidence/relationship matrix (n × q) of genetic effects, a is the vector (u × 1) for additive genetic effects and e is random error. The above REML model was fit separately for each treatment. Rearing tank and group intercept within treatment tank were fit as fixed effects, and animal was the sole random effect. Two groups of three full-sib families having the most extreme mean EBV for growth were selected to represent the 'HIGH' and 'LOW' growth categories, respectively (see Appendix 1)

### Total RNA extraction and reverse transcription

Total RNA was extracted from approximately 25 mg of liver tissue using a Qiazol isolation reagent (RNeasy 96 Universal Tissue, Qiagen), following the manufacturer's instructions. RNA concentrations were determined by spectrophotometry (GeneQuant, Pharmacia). Following DNAse treatment to remove residual genomic DNA, 10 μg total RNA was used in a 100 μl reaction to obtain first-strand cDNA by reverse transcriptase reaction (cDNA Archive Kit, Applied Biosystems).

### Oligonucleotide design and RT-qPCR analysis of gene expression

The mRNA sequences for IGF-1 and GHR were not available for brook charr in EMBL nor NCBI GeneBank [63]. Consequently, we used the mRNA IGF-1 sequence from chum salmon (*O. keta*) (Acc. No. AF063216) and the mRNA GHR sequence from coho salmon (O. *kisutch*) (Acc. No. AF403539) to design non-specific primers. We used these primer pairs to amplify and sequence a region (≈200 bp) of brook charr IGF-1 and GHR mRNA. Gene specific primers and probes were designed from this sequence using Primer Express^® ^software (Table 8). Primers were tested using conventional PCR and tested by amplifying a single band of approximately 90 bp. Primer concentrations were optimized following the manufacturer's instructions (ABI PRISM ^®^7000 Sequence Detection System (SDS), Applied Biosystems). Relative quantification of gene expression was achieved by concurrent amplification of the eukaryotic 18S rRNA endogenous control (Applied Biosystems).

**Table 8:** Primers and probes used in RT-qPCR assays of gene expression.

Target gene	Primer Set (5'→3')	Probe (5'→3')
*IGF-1*	Forward: CAGGCATCCAGATTGTGCAA	CAGCCATTACTCTCTG
	Reverse: ACCATGTTCTGAGAATTCCTGTGTT	
*GHR*	Forward: CCCACTGCCCCCTGTATCT	ACCATGGTGGAAGGAG
	Reverse: CTTCAGAAGGAGGCTGTTTTGC	

Each reaction (25 μl) was run in triplicate and contained 5 μl of cDNA (diluted 1: 10 for target genes and 1: 100 for 18S rRNA gene), 12.5 μl Taqman Universal PCR master mix (Applied Biosystem), and 0.9 μM F/R primers. The thermocycling profile used was the default from the sds 2.0 software (50°C for 2 min, 95°C for 10 min, followed by 40 cycles of 95°C for 15 s and 60°C for 1 min). For each primer and probe set, two negative controls were also amplified: non-reverse transcribed total RNA treated with DNase (as a control for contamination by genomic DNA) and a template negative sample, to control for any contamination of the reagents. Amplification efficiencies for all primer/probe sets were calculated following the manufacturer's instructions, and all values proved to be sufficient to allow direct comparison of amplification plots according to the ΔΔCt method (see Sequence Detection Systems Quantitative Assay Design and Optimization, Applied Biosystems).

### Statistical modeling

All data were modeled under a linear mixed-effects framework (S-Plus 6.1; Insightful Corporation), in which each family was nested as a random factor within their respective EBV group (HIGH/LOW). Thus, the underlying covariance structure inherent within the data set due to non-replicable nesting of one grouping factor within another was accounted for as random variation between EBV group and among families within each EBV group. Effects of rearing environment (saltwater (SW) vs. freshwater (FW)) and sex (male vs. female), and their interactions with genotypic value (HIGH vs. LOW EBV) were modeled as fixed-effects, with parameters estimated by maximum likelihood. Although this analysis captures interaction effects with EBV group, the significance of the main effect of genotypic value cannot directly be estimated from the model due to insufficient (0) degrees of freedom associated with the nesting factor (EBV group). Consequently, the EBV group effect was estimated separately using a linear mixed effects model incorporating random variation among families and fixed-effect differences between EBV groups. Underlying assumptions of normality and homogeneity of variance were evaluated with diagnostic plots. In one case (growth relative to GHR expression; see subsequent section), heteroscedasticity of within group errors was incorporated into the model via separate variance estimators for each grouping stratum.

Model selection followed a backwards step-wise procedure. A maximal model incorporating all simple and interaction terms was initially defined, of the form

yijk = (β0 + bi + bij) + β1E + β2S + β3GxE + β4GxS + β5ExS + β6GxExS + εijk

where yijk is mRNA expression (GHR or IGF-1) or individual absolute growth for the kth individual from the ith family of the kth EBV group, betas (β) corresponding to fixed-effects coefficients for the terms environment (E), sex (S) and EBV group (G), herein also representative of genotypic effects, bi and bij represent random effects vectors describing variation around the model intercepts (i.e. whole-experimental mean) attributable to the ith family nested within the kth EBV group, and εijk is random error. The significance of each term (i.e. model coefficient) was evaluated by conditional F-tests (Table 9, 10, 11). Progressively simpler models were subsequently defined by removing non-significant terms from precedent models beginning with interaction effects. Nested models were then evaluated using likelihood ratio tests to select the most parsimonious model. The reported p-values correspond to results of the likelihood ratio tests wherein a value greater than the nominal level of significance (α = 0.05) indicates that removal of the model term does not increase model deviance significantly [64]. In the event that term removal resulted in a likelihood ratio test approaching a marginal level of significance (0.05 ≤ p ≤ 0.10), final model selection was made through use of the Akaike Information Criterion. Finally, group means were estimated as linear predictors based on the most parsimonious model. Standard errors for each estimate were determined from a bootstrap distribution (1,000 iterations) of model predictions.

**Table 9:** Model selection for absolute growth using a backwards step-wise procedure.

	Absolute growth		
	
Model	Log likelihood	Test	*P*
**Growth **~Env + Sex + EBV·Env + EBV·Sex +Env·Sex +EBV·Env·Sex (1)	-107.460		
~Env + Sex + EBV·Env + EBV·Sex +Env·Sex (2)	-108.443	1 vs. 2	0.169
~Env + Sex + EBV·Env + EBV·Sex (3)	-108.499	2 vs. 3	0.737
**~Env + Sex + EBV·Env (4)**	**-108.607**	**3 vs. 4**	**0.643**
~Env + Sex (5)	-112.465	4 vs. 5	0.021

Progressively simpler models are subsequently defined by removing terms from previous models beginning with interaction effects (*e.g*. 1 vs. 2). Nested models were tested evaluated using likelihood ratio tests to select the most parsimonious model. The reported *p*-values correspond to results of the likelihood ratio tests wherein a value greater than the nominal level of significance (α = 0.05) indicates that removal of the model term does not increase model deviance significantly. The final, parsimonious model that was selected is indicated in bold characters (Env = environment, EBV = EBV group).

**Table 10:** Model selection for gene expression using a backwards step-wise procedure.

	GHR			IGF-1		
		
Model	Log likelihood	Test	*P*	Log likelihood	Test	*P*
**Gene **~Env + Sex + EBV·Env + EBV·Sex +Env·Sex +EBV·Env·Sex (1)	-82.617			-198.918		
~Env + Sex + EBV·Env + EBV·Sex +Env·Sex (2)	-83.111	1 vs. 2	0.320	-199.142	1 vs. 2	0.502
~Env + Sex + EBV·Env + EBV·Sex (3)	-83.460	2 vs. 3	0.403	-199.170	2 vs. 3	0.815
~**Env + Sex + EBV·Env (4)**	**-84.513**	**3 vs. 4**	**0.147**	**-200.233**	**3 vs. 4**	**0.145**
~Env + Sex (5)	-87.055	4 vs. 5	0.079	-203.634	4 vs. 5	0.033

Progressively simpler models are subsequently defined by removing terms from previous models beginning with interaction effects (e.g. 1 vs. 2). Nested models were tested evaluated using likelihood ratio tests to select the most parsimonious model. The reported p-values correspond to results of the likelihood ratio tests wherein a value greater than the nominal level of significance indicates that removal of the model term does not increase model deviance significantly. The final, parsimonious model that was selected is indicated in bold characters (Env = environment, EBV = EBV group). In the case of GHR, removal of the EBVxEnv term (model 5) resulted in a marginally non-significant likelihood ratio test. However, the interaction term was retained in the final model (model 4 AIC = 185.02), given that its exclusion resulted in a greater penalised likelihood score (model 5 AIC = 186.11).

**Table 11:** Model selection for individual transcript efficiency using a backwards step-wise procedure.

	iTE_GHR			iTE_IGF-1		
		
Model	Log likelihood	Test	*P*	Log likelihood	Test	*P*
iTE ~Env + Sex + EBV·Env + EBV·Sex +Env·Sex +EBV·Env·Sex (1)	-297.317			-436.651		
~Env + Sex + EBV·Env + EBV·Sex +Env·Sex (2)	-297.689	1 vs. 2	0.388	-436.657	1 vs. 2	0.906
~Env + Sex + EBV·Env + EBV·Sex (3)	-297.945	2 vs. 3	0.474	-436.897	2 vs. 3	0.489
~**Env + Sex + EBV·Env (4)**	**-298.417**	**3 vs. 4**	**0.331**	**-437.236**	**3 vs. 4**	**0.410**
~Env + Sex (5)	-302.856	4 vs. 5	0.012	-439.672	4 vs. 5	0.088

Progressively simpler models are subsequently defined by removing terms from previous models beginning with interaction effects (e.g. 1 vs. 2). Nested models were tested evaluated using likelihood ratio tests to select the most parsimonious model. The reported p-values correspond to results of the likelihood ratio tests wherein a value greater than the nominal level of significance (α = 0.05) indicates that removal of the model term does not increase model deviance significantly. The final, parsimonious model that was selected is indicated in bold characters (Env = environment, EBV = EBV group). In the case of iTE_IGF-1_, removal of the EBVxEnv term (model 5) resulted in a marginally non-significant likelihood ratio test. However, the interaction term was retained in the final model (model 4 AIC = 890.47), given that its exclusion resulted in a greater penalised likelihood score (model 5 AIC = 891.34).

### Individual transcript efficiency (iTE)

Given the direct role of both IGF-1 and GHR in the physiological process of vertebrate growth [16,18-20,27,29,51], the effect of environmental modification on their ultimate phenotypic expression might be most effectively surveyed from the direct ratio of gram-to-gain for inferred mRNA production to phenotype. Such an analysis would reflect that of a simple single-QTL design or candidate gene analysis without control for physiological/genomic background. Here, mRNA production was inherently evaluated relative to that of an endogenous control molecule (eukaryotic 18S rRNA; see above), which at the least controls for gross effects of general physiological background. Thus, in order to evaluate relative association between mRNA production and phenotype, we propose to measure a ratio termed 'individual transcript efficiency' (iTE). This iTE ratio represents the effective growth per unit of mRNA production (GA/X in g·d^-1^·u^-1^, where GA is the absolute growth and X is the production in units of IGF-1 or GHR (units is arbitrary, herein the value representing the fold difference between a given gene and individual control). We tested iTE for GHR and IGF-1 using the same general model (see above under Statistical modeling) to evaluate effects associated with integral genetic value (families from either HIGH or LOW growth category), saline environment (FW/SW), sex and GxE interaction on the relative genomic efficiency for each gene, i.e. the dependence of their specific capacity to produce phenotype depending on environmental and genetic group effects.

## Authors' contributions

GC participated in the design of the study, was responsible for collecting samples, carried out the molecular work, drafted and wrote the manuscript. GP participated in the design of the study and the collection of the samples, participated in the analysis and interpretation of data, and contributed to writing the manuscript. PB commented on an earlier draft of the paper. LB initiated the idea of testing GxE interactions at the gene transcription level, participated in the design of the study and the interpretation of data, and contributed to writing the manuscript. All authors read and approved the final manuscript.
